# Supplementary material for: Wood Formation in Trees Is Increased by Manipulating PXY-Regulated Cell Division
Source: Curr Biol. 2015 Apr 20;25(8):1050–5. doi: 10.1016/j.cub.2015.02.023 (PMC4406943; doi:10.1016/j.cub.2015.02.023)
Supplement: Document S1. Supplemental Experimental Procedures, Figures S1–S4, and Table S1 [file mmc1.pdf]

Current Biology

Supplemental Information

Wood Formation in Trees Is Increased by Manipulating PXY-Regulated Cell Division

J. Peter Etchells, Laxmi S. Mishra, Manoj Kumar, Liam Campbell, and Simon R. Turner

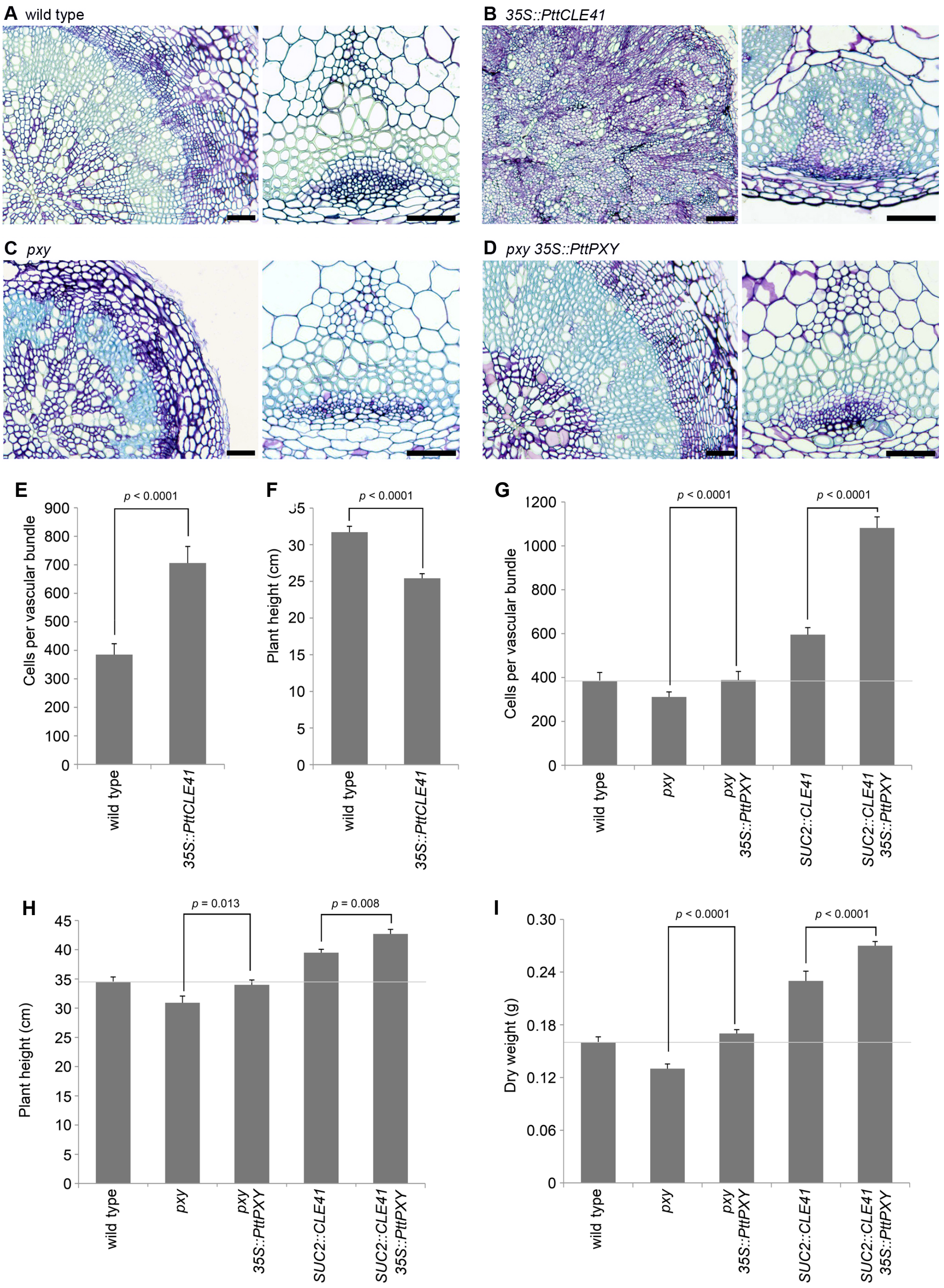

Figure S1: Poplar *CLE41* and *PXY* genes are functional in *Arabidopsis*.
(**A-D**) Sections from *Arabidopsis* hypocotyls (LHS) and inflorescence stem vascular bundles (RHS) from typical wild type (**A**), *35S::PttCLE41* (**B**), *pxy* mutant (**C**), and a *pxy* mutant complemented with *35S::PttPXY* (**D**). **(E-I)** Growth characteristics of *Arabidopsis* lines overexpressing poplar *PXY* genes. Number of cells per vascular bundle (**E**) and plant height (**F**) of *Arabidopsis 35S::PttCLE41* lines compared to wild type counterparts. Number of cells per vascular bundle (**G**), plant height (**H**) and dry weight (**I**) of *Arabidopsis pxy*, *pxy 35S::PttPXY*, *SUC2::AtCLE41*, *SUC2::AtCLE41-35S::PttPXY* lines compared to wild type counterparts. P values were calculated with an ANOVA and LSD post-hoc test with N = 10 (E,F) or 40 (G,H,I). Scales bars indicate 50 µM (A-D). Error bars indicate the standard error. Related for Figure 1.

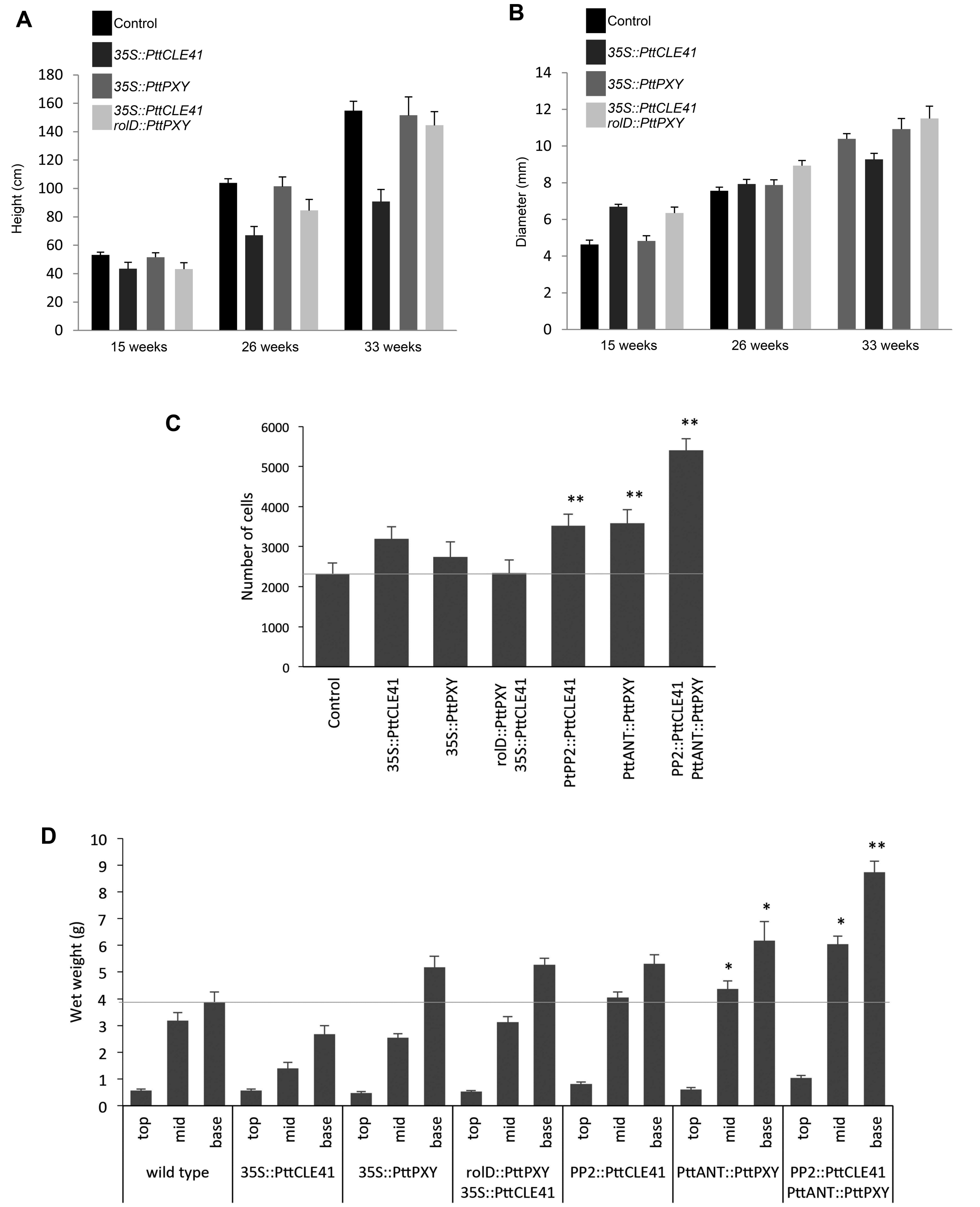

Figure S2: Growth characteristics of aspen lines overexpressing *PttCLE41/PttPXY*. Height (**A**) and diameter (**B**) measurements from hybrid aspen grown in soil. Trees rooted in April, were measured at 15 weeks (July), 26 weeks (August) and 33 weeks (October). (**C**) Graph showing number of vascular cells in control and *35S::PttCLE41*, *35S::PttPXY*, 35S::*PttCLE41 rolD::PttPXY*, *PtPP2::PttCLE41*, *PttANT::PttPXY* and *PtPP2::PttCLE41 PttANT::PttPXY* hybrid aspen lines in tissue culture 3 weeks post-rooting. (**D**) Wet weight of 10 cm pieces of sapling stem taken from the base, middle (50th internode) and top, except for *35S::PttCLE41* that had less than 50 internodes and sections were taken midway between the top and bottom instead. Graph shows the wet weight of stem pieces in Fig. 4G. *Significantly larger than wild type $p < 0.05$; ** Significantly larger than all other lines $p < 0.001$; values were calculated with an ANOVA and LSD post-hoc test, A-B, n = 15; C-D, n=7. Error bars indicate the standard error. Related to Figure 3.

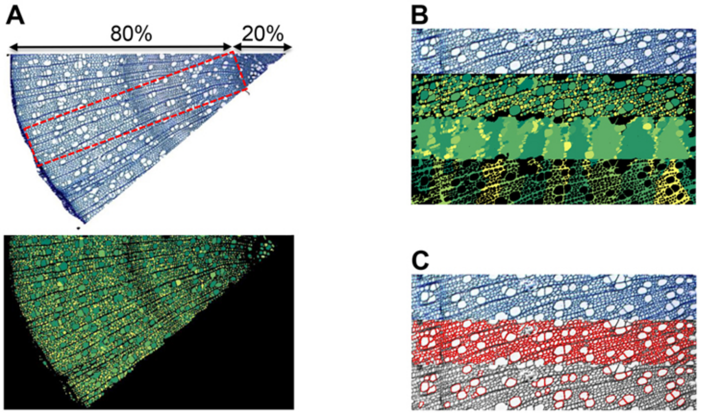

Figure S3: Phenotypic characterisation using Cellprofiler.
(**A**) Transverse stem section from the 50th internode showing xylem in a sector with a central angle of 40° (top) and recognition of cell lumens by Cellprofiler (below). Cells were identified with greater than 95% accuracy, but cells with no clear lumen, such as ray cells or very small fiber cells, were not recognized. (**B**) Measurement of cell size and cell wall area is based upon a rectangle (top) outlined in (a). Primary objects (cell lumens) were identified (upper middle) and propagated outwards to identify the secondary objects (lower middle). The tertiary objects (cell walls) were obtained by subtracting the primary objects from the secondary objects (bottom). (**C**) Identification of vessels based upon identifying primary objects (middle) and then filtering by size and shape (bottom). Relates to the supplementary experimental procedures.

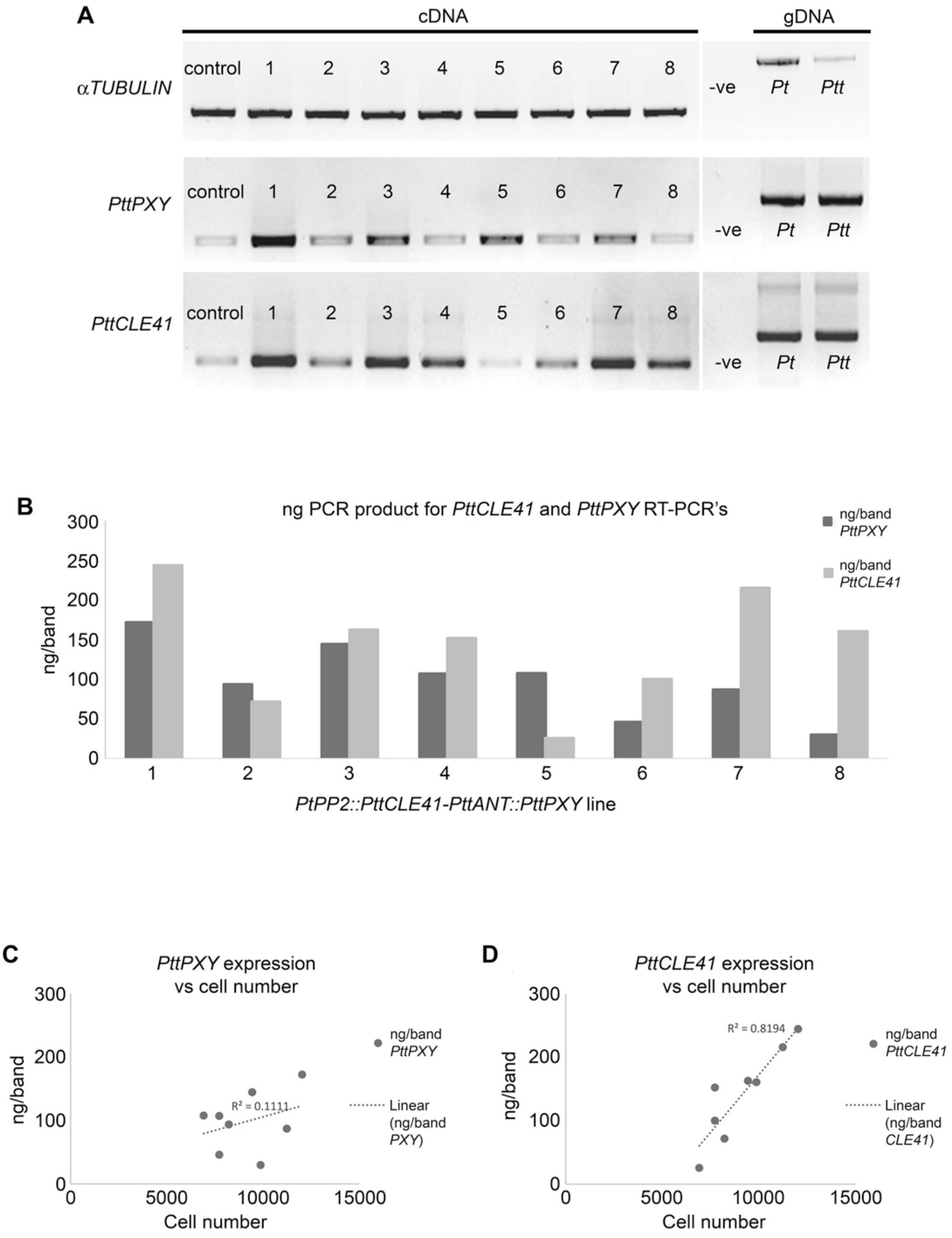

Figure S4: *PttCLE41* and *PttPXY* expression analysis in *PtPP2::PttCLE41-PttANT::PttPXY* lines.
(**A**) RT-PCR showing expression in 8 independent transgenic lines. Stem material was taken adjacent to the 50th internode. (**B**) Relative intensity of PCR product in (A), was determined using Image Lab 5.1 software (Bio-rad). (**C**) Relationship between cell number and *PttPXY* expression. (**D**) Relationship between cell number and *PttCLE41* expression. Related to Figure 4.

Table S1: Analysis of transverse section of xylem from the 50th internode of control and *PtPP2::CLE41-PttANT::PXY* plants.

	Control	*PtPP2::CLE41-PttANT::PXY*
Average cell size	607 ± 13	577 ± 19
Average lumen size	312 ± 13	346 ± 9
Average cell wall area	265 ± 24	260 ± 16
Vessels per 1000 cells	50 ± 2	49 ± 6

Mean of 5 independent lines are shown with the standard error. Area measurements are in arbitrary units. Statistical analysis was carried out using a T-test, no significant differences were found. Related to Figure 4.

Supplementary Experimental Procedures

Binary vectors for plant transformation

For poplar 35S overexpression vectors, primers were designed against *Populus trichocarpa* [S1] *CLE41* (Potri.012G019400) (PttCLE41-F: CACCTAGCTAGCCTTGGTGCTGGT, PttCLE41-R ACCCCTTAATTCCCCCATTA) and *PXY* (Potri.003G107600) (PttPXY-F:CACCATGAAACTCCCTTTTCTTTT, PttPXY-R ACATTCGACTGCAGGCTTTT) and used to amplify sequences from DNA extracted from hybrid aspen (*Populus tremula x tremuloides* clone T89). These were subsequently cloned into pK2GW7 [S2] via pENTR-D-TOPO. For the *rolD::PttPXY 35S::CLE41* construct, *PttCLE41* was subcloned into pDONRP4-P3 which was combined with *pENTR-D-TOPO-PttPXY* and *pK7m34GW2-8m21GW3* [S3] using an LR clonase reaction. Cloned *PttPXY* and *PttCLE41* were sequenced either in entry clones or expression clones. Sequences were annotated by aligning with *P. trichocarpa* sequences. Annotated sequences for *PttPXY* (accession number, KP682331) and *PttCLE41* (accession number, KP682332) were submitted to NCBI. During the cloning a mutation was accidently introduced that had removed the stop codon at the end of the *PttPXY* gene and resulted in a 44 amino acid extension encoded by the vector being added to the C terminus.

For tissue specific expression, *PttPXY* and *PttCLE41* pENTR-D/TOPO entry clones were used in an LR clonase reaction in combination with custom Gateway destination vectors, pVX31 (*Apa*I-p*PttANT1*-*Spe*I-R1R2 Gateway Cassette-t35S-*Sbf*I) and pVX33 (*Sbf*I-p*PtPP2*-*Spe*I-R1R2 Gateway Cassette- t35S-*Sbf*I), which were constructed in a pCambia2300 backbone using restriction based cloning. The promoter sequences were chosen on the basis of poplar expression data. *PttANT* (Potri.002G114800) regulatory sequences were used for cambium specific expression and a *PtPP2* (Potri.015G120200) promoter was used for phloem specific expression. For the *PtPP2* promoter from *Populus trichocarpa* primers (pPtPP2-F atccctaggcctgcaggTAAGCTATGTACGTTTTGG, pPttANT-R atcactagtGACAAGCTGAGAGACTG) were used to amplify a fragment of 1999 bp upstream of the start codon. For the *PttANT1* promoter, primers (pPttANT-F atcgggcccCCGAAGTTGCTCACTTC, pPttANT-R atcactagtGACAAGCTGAGAGACTG) were used to amplify a sequence 1156 bp upstream of start codon to 904 bp downstream of the start codon that drove expression in vascular tissue. To create the double tissue specific expression construct, *Sbf*I-*pPtPP2-PttCLE41-* t35S-*Sbf*I cassette was excised and cloned into *Sbf*I site of *Apa*I-*pPttANT1-PttPXY*-t35S-*Sbf*I.

Transcriptional reporter lines for p*PttANT* and p*PtPP2* were generated by cloning a fragment encoding eGFP-GUS in in pVX31 and pVX33 using an LR clonase reaction resulting in p*PttANT::eGFP-GUS* and p*PtPP2::eGFP-GUS* constructs. Hand sections were stained using a variation on the method described in Rodrigues- Pousada [S4] and were viewed following clearing by overnight incubation at 4°C in chloral hydrate solution [S5].

Plant transformation

Arabidopsis transformation was carried out using the method of Clough and Bent [S6]. For transformation of hybrid aspen (clone T89), a method based on that of Nilsson et al [S7] was used. Briefly, *Agrobacterium* strain GV3101 harbouring a binary vector was grown to an OD_{600} of 0.6, collected by centrifugation in a 50 ml tube and resuspended in MS medium, pH5.8 supplemented with acetosyringone to a final concentration of 25 μM at room temperature. Leaf and petiole sections were cut

from hybrid aspen grown under sterile conditions and incubated in the resuspended *Agrobacterium* for 1 hour and placed on MS agar supplemented with 2% sucrose, BAP (0.2 mg/L), IBA 0.1 mg/L and TDZ (0.1 mg/L) prior to incubation in dark for 48 hours. Subsequently, plant pieces were rinsed in MS and placed in the light on MS agar supplemented with 250 µg/ml cefatoxime and 100 µg/ml kanamycin. Following shoot initiation calli were transferred to woody plant medium (WPM) supplemented with sucrose (2%), BAP (0.2 mg/L), IBA (0.1 mg/L), kanamycin (100 µg/ml) for shoot elongation and subsequently to WPM for rooting.
In order to synchronise plant growth for subsequent analysis the top 2 cm of each plantlet to be used was removed and re-rooted on the same day. All plants used for growth analysis were grown side by side in the same incubator and transferred to soil on the same day once roots were established. For long term growth, plants were transferred to a greenhouse in April and maintained for up to 12 months.

Determination of plant growth characteristics
Vascular organization was determined using plant material fixed in FAA, dehydrated through an ethanol series before infiltration and embedding with JB4 embedding media. 5 µM sections were stained with 0.05% aqueous toluidine blue, mounted in Cytoseal and visualised on a Leica 5500 microscope. Vascular tissue was considered to be ordered if xylem could be incorporated in an elliptical shape that excluded the phloem. Xylem cell counts and determination of cell wall area was performed on cross sections from the entire cross section of tissue culture plants 3 weeks post rooting or from the 50th internode of greenhouse grown plants in which case only a 40° segment of the stem was used (Figure S3A). Cell counting was carried out using Cellprofiler [S8] as outlined in Figure S3. For greenhouse grown plants, 10cm segments were sampled from base of the plant, from 50th internode and from the top of the plants, 12 months following transfer to soil. Material was dried at 50°C for 4 weeks before weighing.

Supplementary references

S1. Tuskan, G.A., DiFazio, S., Jansson, S., Bohlmann, J., Grigoriev, I., Hellsten, U., Putnam, N., Ralph, S., Rombauts, S., Salamov, A., et al. (2006). The Genome of Black Cottonwood, Populus trichocarpa (Torr. & Gray). Science *313*, 1596-1604.
S2. Karimi, M., Inze, D., and Depicker, A. (2002). GATEWAY(TM) vectors for Agrobacterium-mediated plant transformation. Trends in Plant Science *7*, 193-195.
S3. Karimi, M., Bleys, A., Vanderhaeghen, R., and Hilson, P. (2007). Building Blocks for Plant Gene Assembly. Plant Physiology *145*, 1183-1191.
S4. Rodrigues-Pousada, R.A., De Rycke, R., Dedonder, A., Van Caeneghem, W., Engler, G., Van Montagu, M., and Van Der Straeten, D. (1993). The Arabidopsis 1-Aminocyclopropane-1-Carboxylate Synthase Gene 1 Is Expressed during Early Development. The Plant Cell Online *5*, 897-911.
S5. Berleth, T., and Jurgens, G. (1993). The role of the monopteros gene in organising the basal body region of the Arabidopsis embryo. Development *118*, 575-587.
S6. Clough, S.J., and Bent, A.F. (1998). Floral dip: a simplified method for Agrobacterium-mediated transformation of Arabidopsis thaliana. Plant Journal *16*, 735-743.
S7. Nilsson, O., Aldén, T., Sitbon, F., Anthony Little, C.H., Chalupa, V., Sandberg, G., and Olsson, O. (1992). Spatial pattern of cauliflower mosaic

virus 35S promoter-luciferase expression in transgenic hybrid aspen trees monitored by enzymatic assay and non-destructive imaging. Transgenic Research *1*, 209-220.

S8. Carpenter, A.E., Jones, T.R., Lamprecht, M.R., Clarke, C., Kang, I.H., Friman, O., Guertin, D.A., Chang, J.H., Lindquist, R.A., Moffat, J., et al. (2006). CellProfiler: image analysis software for identifying and quantifying cell phenotypes. Genome Biol *7*, R100.
