# Supplementary material for: Wood Formation in Trees Is Increased by Manipulating PXY-Regulated Cell Division
Source: Curr Biol. 2015 Apr 20;25(8):1050–5. doi: 10.1016/j.cub.2015.02.023 (PMC4406943; doi:10.1016/j.cub.2015.02.023)
Supplement: Document S2. Article plus Supplemental Information [file mmc2.pdf]

Report

Current Biology

Wood Formation in Trees Is Increased by Manipulating PXY-Regulated Cell Division

Graphical Abstract

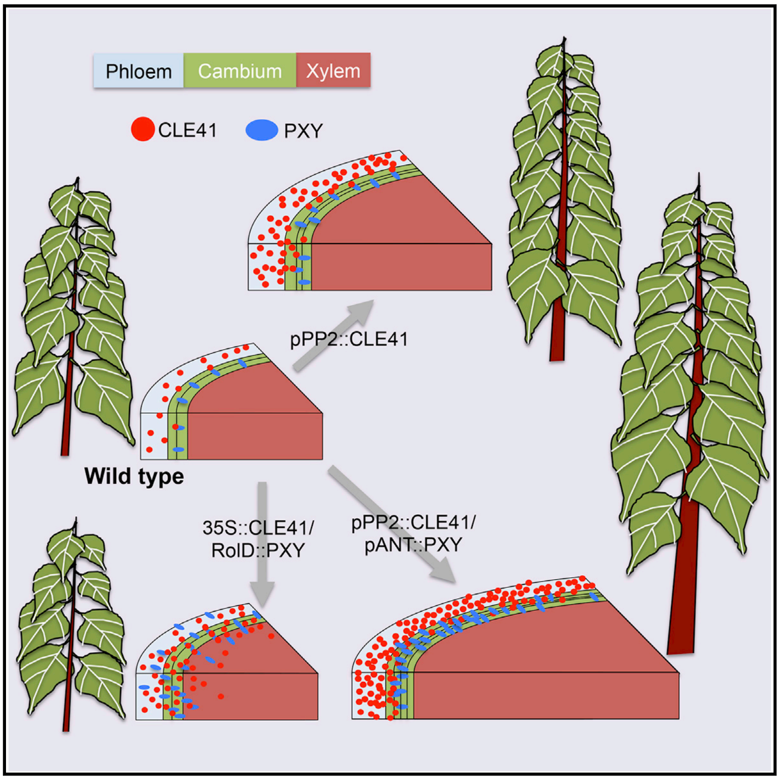

Highlights

- PXY receptor kinase and CLE peptide signal to regulate radial growth in trees
- Engineering PXY-CLE expression can lead to large increases in wood formation
- Manipulating PXY-CLE also leads to increased tree height and leaf size
- The results can be used to generate trees that are more productive

Authors

J. Peter Etchells, Laxmi S. Mishra, ..., Liam Campbell, Simon R. Turner

Correspondence

simon.turner@manchester.ac.uk

In Brief

Etchells et al. show that altering the expression of the poplar homologs of the receptor kinase *PXY* and its peptide ligand *CLE41* results in increased cambial cell division in hybrid aspen. 2-fold increases in the rate of wood formation were observed, demonstrating that engineering PXY/CLE41 signaling offers a means to increase tree productivity.

Accession Numbers

KP682331
KP682332

Etchells et al., 2015, Current Biology *25*, 1050–1055
April 20, 2015 ©2015 The Authors
http://dx.doi.org/10.1016/j.cub.2015.02.023

CellPress

Current Biology
Report

Wood Formation in Trees Is Increased by Manipulating PXY-Regulated Cell Division

J. Peter Etchells,[1,2,3] Laxmi S. Mishra,[1,3] Manoj Kumar,[1] Liam Campbell,[1] and Simon R. Turner[1,*]
[1]Faculty of Life Sciences, University of Manchester, Oxford Road, Manchester M13 9PT, UK
[2]Present address: School of Biological and Biomedical Sciences, Durham University, South Road, Durham DH1 3LE, UK
[3]Co-first author
*Correspondence: simon.turner@manchester.ac.uk
http://dx.doi.org/10.1016/j.cub.2015.02.023
This is an open access article under the CC BY license (http://creativecommons.org/licenses/by/4.0/).

SUMMARY

The woody tissue of trees is composed of xylem cells that arise from divisions of stem cells within the cambial meristem. The rate of xylem cell formation is dependent upon the rate of cell division within the cambium and is controlled by both genetic and environmental factors [1, 2]. In the annual plant *Arabidopsis*, signaling between a peptide ligand CLE41 and a receptor kinase PXY controls cambial cell divisions [3–5]; however, the pathway regulating secondary growth in trees has not been identified. Here, we show that an aspen receptor kinase PttPXY and its peptide ligand PttCLE41 are functional orthologs and act to control a multifunctional pathway that regulates both the rate of cambial cell division and woody tissue organization. Ectopic overexpression of *PttPXY* and *PttCLE41* genes in hybrid aspen resulted in vascular tissue abnormalities and poor plant growth. In contrast, precise tissue-specific overexpression generated trees that exhibited a 2-fold increase in the rate of wood formation, were taller, and possessed larger leaves compared to the controls. Our results demonstrate that the PXY-CLE pathway has evolved to regulate secondary growth and manipulating this pathway can result in dramatically increased tree growth and productivity.

RESULTS AND DISCUSSION

The PXY-CLE Signaling Pathway Is Conserved in Trees and Acts to Regulate Secondary Growth

Wood is composed of xylem cells that arise from divisions of stem cells that reside within the vascular meristem, known as the cambium or procambium. One mechanism that promotes cell division in vascular meristems of *Arabidopsis* involves phloem-specific expression of *CLE41* that encodes a peptide ligand known as TDIF. TDIF is perceived by a receptor kinase, PXY (also known as TDR), that is expressed in the adjacent stem cells of the procambium [3–6]. PXY controls both the orientation [3, 4] and rate of cell division in procambial stem cells [7, 8] and inhibits their differentiation into xylem [5, 9]. Consequently, while ectopically overexpressing *CLE41* in *Arabidopsis* increases the number of cells in vascular bundles, these increases are accompanied by repression of xylem differentiation and loss of vascular organization [3, 5, 10]. Furthermore, output from the pathway is regulated by a negative feedback loop in which *CLE41* expression results in downregulation of *PXY* [3]. To determine whether PXY-CLE41 signaling is conserved in poplar, we cloned putative orthologs of *PXY* and *CLE41* genes from the hybrid aspen (*Populus tremula* × *P. tremuloides*), referred to hereafter as *PttPXY* and *PttCLE41*, respectively. When overexpressed in *Arabidopsis*, *35S::PttCLE41* lines demonstrated a loss of vascular organization, increased numbers of cells per vascular bundle, and decreased plant height (Figures S1A, S1B, S1E, and S1F). The *35S::PttPXY* construct complemented the *Arabidopsis pxy* mutant phenotype (Figures S1C, S1D, S1G, and S1H), and this complemented line also restored the ability of the plants to respond to overexpression of the *AtCLE41* ligand (Figures S1G and S1H). As such, both *PttCLE41* and *PttPXY* clones act as functional orthologs of their respective *Arabidopsis* genes. Furthermore, expression of *PttPXY* in *Arabidopsis* plants already engineered for tissue-specific *AtCLE41* overexpression resulted in increased plant biomass (Figure S1I).

Ectopic Expression of *PttCLE41* or *PttPXY* Leads to Abnormal Vascular Tissue Development in Trees

We investigated the consequence of constitutively overexpressing these genes in trees by making use of the 35S promoter that is known to give widespread expression in hybrid aspen [11]. We used our *35S::PttPXY* and *35S::PttCLE41* constructs (see above) individually or overexpressed both genes together in a single binary plasmid that contained *35S::PttCLE41* and *rolD::PttPXY* cassettes. To varying degrees, all independent lines (n = 15) of *35S::PttCLE41* hybrid aspen had intercalated xylem and phloem (Figure 1A). *35S::PttPXY* lines (n = 10) also demonstrated disrupted organization in parts of the xylem, but to a much lesser extent than seen in *35S::PttCLE41* (Figure 1A). 7 out of 15 *35S::PttCLE41-rolD::PttPXY* lines appeared normal, whereas the remaining 8 exhibited varying degrees of tissue disruption (Figure 1A). None of these lines led to significant increases in tree growth; in fact, *35S::PttCLE41* lines were significantly shorter than wild-type (Figures S2A and S2B), exhibiting various growth abnormalities (Figure 1B).

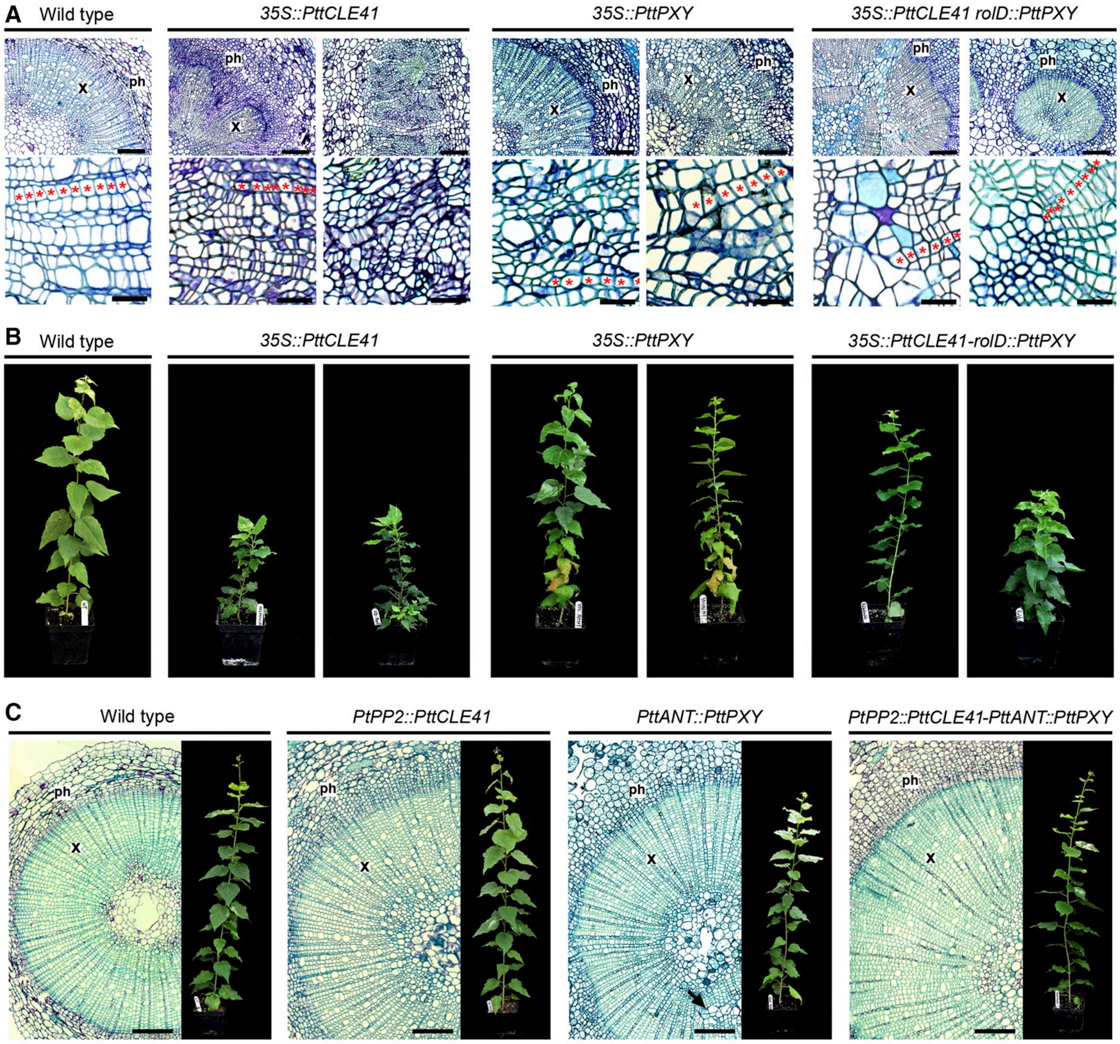

Figure 1. Phenotypes of Hybrid Aspen Ectopically Overexpressing *PttCLE41* and/or *PttPXY* Genes

(A) Sections from tissue-culture-grown plantlets 3 weeks post-rooting. Where two images are shown in the upper panel, they were selected to show the range of phenotypes observed. Scale bars represent 200 μM (upper panels) and 50 μM (lower panels). Red asterisks show examples of organized files of cells. The xylem (x) and phloem (ph) are indicated.

(B) Representative greenhouse-grown plants 3 months after transfer to soil.

(C) Phenotypes of hybrid aspen with targeted overexpression of *PttCLE41* and *PttPXY*. Left-hand panels show sections from tissue-culture-grown plantlets 3 weeks post-rooting while greenhouse-grown plants 3 months after transfer to soil are shown on the right. Scale bars represent 200 μM. The xylem (x) and phloem (ph) are also indicated. Arrows highlight the disrupted xylem. See also Figure S1.

Tissue-Specific Expression of *PttPXY* and *PttCLE41* Increases Vascular Cell Division and Retains Normal Vascular Tissue Organization

We hypothesized that the tissue-specific expression of both *PttPXY* and *PttCLE41* might be important both for tissue organization and for maximizing cambial cell division. Transcriptomic data show that in poplar, *PXY* is expressed predominantly in the cambium and at a low level in the xylem [12]. Poplar microarray data identified the *ANTEGUMENTA* (*ANT*) gene as highly expressed only within the division zone [12]. Using an early draft of the *Populus trichocarpa* genome [13] as a guide, we identified and cloned a putative promoter from hybrid aspen (*PttANT*), although better annotation of the genome subsequently suggested the *PttANT* promoter fragment contained sequences both upstream and downstream of the putative transcriptional start site. Analysis of leaves from *PttANT::GUS* plants showed clear vascular-specific GUS expression, while in the stems, GUS activity was restricted to the dividing cambial zone (Figure 2B), consistent with our initial interpretation of the expression data. We also identified and cloned regulatory sequences from a phloem-specific lectin gene, *PHLOEM PROTEIN2* (*PP2*), from *Populus trichocarpa* (*PtPP2*). GUS analysis verified this promoter as vascular tissue-specific in the leaves and giving excellent phloem-specific expression in stems (Figure 2C). These promoters were used to generate three constructs designed to give tissue-specific increases in expression: *PttANT::PttPXY*,

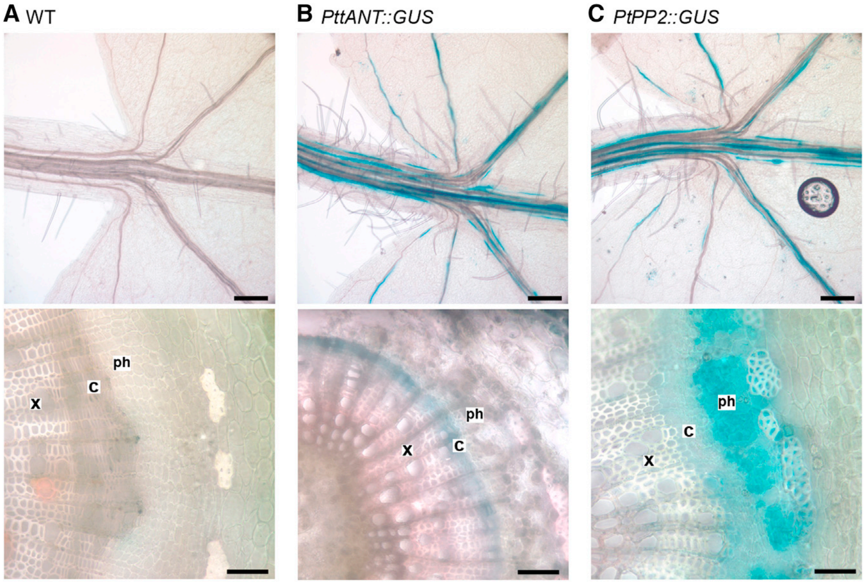

Figure 2. Expression Patterns Derived from *PttANT* and *PtPP2* Promoters

(A–C) GUS-stained and cleared control (A), *PttANT::GUS* (B), and *PtPP2::GUS* (C) plant material. Upper panels show leaves; lower panels are transverse stem sections. Scale bars represent 200 μm (upper panels) and 100 μm (lower panels).

PtPP2::PttCLE41, and *PtPP2::PttCLE41-PttANT::PttPXY*. In contrast to *35S::PttCLE41* (Figure 1A), *PtPP2::PttCLE41* lines demonstrated highly organized vasculature in all 14 lines examined (Figure 1C). 7 out of 15 *PttANT::PttPXY* lines demonstrated minor disruptions in xylem morphology (Figure 1C, arrow) similar to those observed in *35S::PttPXY* trees (Figure 1A); however, all 12 independent *PtPP2::PttCLE41-PttANT::PttPXY* double overexpression lines analyzed exhibited highly organized vascular tissue comparable to that of wild-type controls (Figure 1C). Strikingly, *PtPP2::PttCLE41*, *PttANT::PttPXY*, and *PtPP2::PttCLE41-PttANT::PttPXY* double overexpression lines clearly demonstrated increases in the number of vascular cells as early as 3 weeks post-rooting in tissue culture (Figure S2C).

Tissue-Specific Expression of *PttPXY* and *PttCLE41* Results in Trees that Grow Faster

We further monitored the growth of these transgenic hybrid aspen trees following transfer to soil and maintenance in the greenhouse. Over a 6-month period, *PtPP2::PttCLE41*, *PttANT::PttPXY*, and *PtPP2::PttCLE41-PttANT::PttPXY* plants grew normally (Figure 1C) and were consistently larger than the control plants, with both greater stem diameter and plant height (Figures 3A and 3B). *PtPP2::PttCLE41-PttANT::PttPXY* lines gave the largest increase in radial growth, and after 6 months in the greenhouse, they exhibited a 35% increase in stem diameter compared to untransformed controls and a 10% increase compared to *PtPP2::PttCLE41*, the next best-performing genotype (Figure 3A). The *PtPP2::PttCLE41-PttANT::PttPXY* lines also demonstrated a 56% increase in height over their wild-type counterparts and a 12% increase in height over the next best-performing transgenic line (*PttANT::PttPXY*) (Figure 3B). This increase was due to a generally faster growth rate, with *PtPP2::PttCLE41-PttANT::PttPXY* plants having on average 90 internodes compared to a mean of 60 for control plants (Figure 3C), as well as to an increase in internode length (Figure 3D). While the plants appeared morphologically normal (Figure 1C), the *PtPP2::PttCLE41-PttANT::PttPXY* lines also exhibited increases in leaf area (Figure 3E), with the average leaf area increasing by almost 2-fold. These increases in growth may reflect PXY/CLE signaling acting on other aspects of plant development or be a consequence of increases in sink strength. Although further work is needed to test these hypotheses and to understand the basis of these developmental changes, they do contribute to a general increase in biomass that is likely to further improve the effectiveness of any biotechnological application of these discoveries.

Tissue-Specific Expression of *PttPXY* and *PttCLE41* Results in Large Increases in Wood and Biomass Formation

To better understand the cause of the increases in stem diameter in *PtPP2::PttCLE41-PttANT::PttPXY* lines, at 33 weeks, we harvested half of the trees from each line and sectioned stem material in order to perform cell counts for each line as described in Figure S3. In order to examine material from a similar developmental stage and to account for the differing sizes of the trees examined, we carried out the analysis on material from the 50th internode. We observed a dramatic increase in xylem cell numbers that correlated with the increase in stem diameter, with *PtPP2::PttCLE41-PttANT::PttPXY* lines having the largest number of xylem cells, 189% that of control plants (Figure 3F). Within individual lines, there was also a correlation between cell numbers and *PttCLE41* expression and, to a lesser extent, with *PttPXY* expression (Figure S4). To determine whether it was possible to increase wood formation without altering xylem morphology, we adapted Cellprofiler [14] to measure a number of morphological characteristics of the xylem (Figure S3). The analysis revealed no significant differences in average cell size, average cell lumen size, average cell wall area, and vessel numbers as a proportion of total xylem cells in *PtPP2::PttCLE41-PttANT::PttPXY* compared to controls lines (Table S1), indicating that the increased wood production did not alter wood morphology.

To determine whether the improved growth characteristics led to increased woody biomass, we allowed the remaining trees to grow for an additional 6-month period, after which we determined dry weight (Figure 3G) and wet weight (Figure S2D) at various points along the stem. Consistent with our previous observations, measurement at the base, at the 50th internode (middle), and at the top of the stem demonstrated that *PtPP2::PttCLE41-PttANT::PttPXY* lines exhibited significant

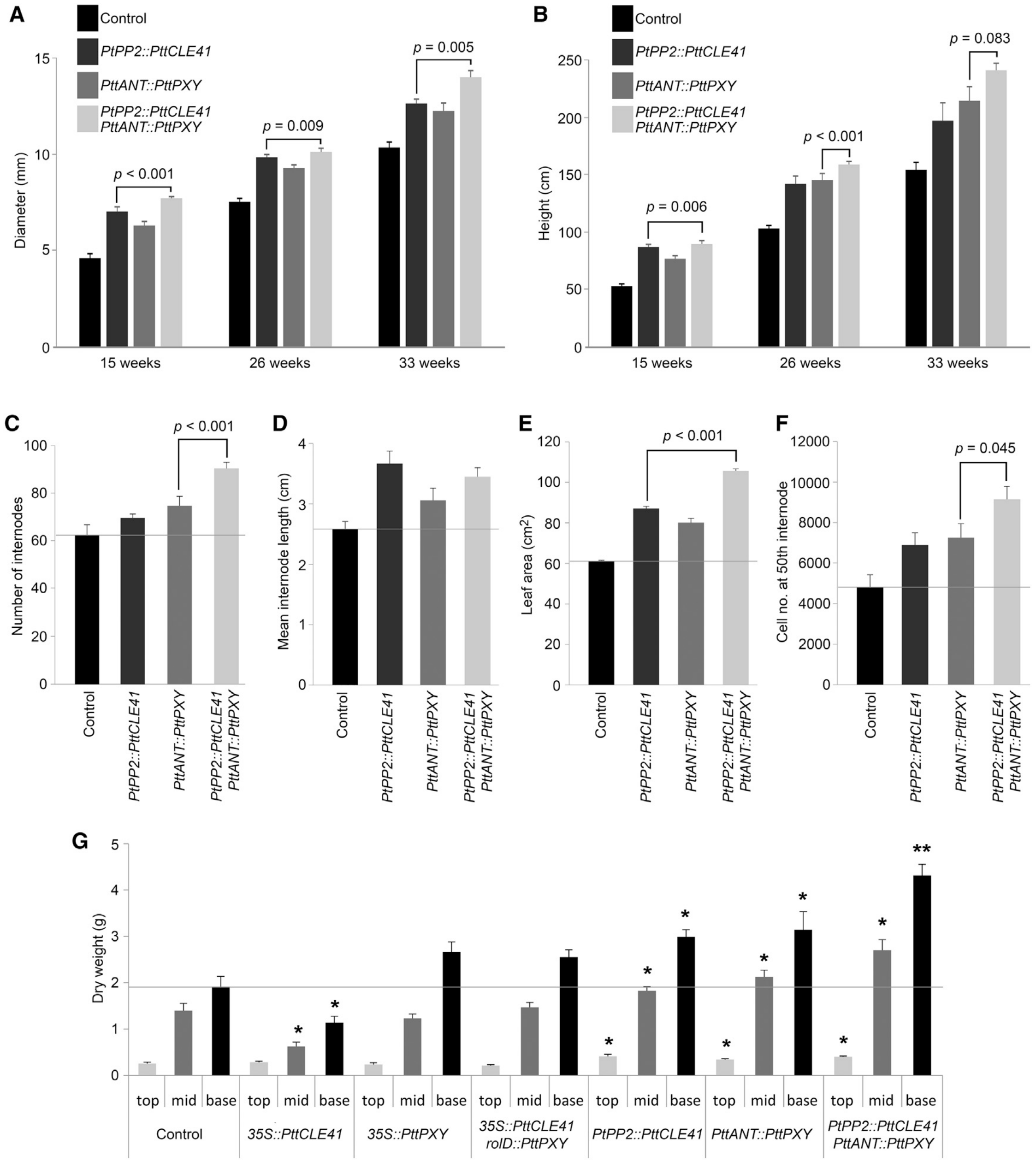

Figure 3. Growth Characteristics of Trees with Targeted *PttCLE41/PttPXY* Overexpression

(A and B) Mean stem diameter (A) and plant height (B) measurements from hybrid aspen grown in soil are shown. Trees rooted in April were measured at 15 weeks (July), 26 weeks (August), and 33 weeks (October).

(C–F) Further analysis of 6-month-old plants: number of internodes (C), length of 50th internode (D), leaf area calculated from measurements of five leaves from around the 50th internode (E), and xylem cell number in a sector, with a central angle of 40°, of a stem transverse section taken from the 50th internode (F).

(G) Graph showing dry weight of 10-cm pieces of sapling stem. Samples were taken from the base, middle (50th internode), and top, except for *35S::PttCLE41*, which had less than 50 internodes and a section taken midway between the top and bottom was used instead.

All p values were calculated with an ANOVA and a least significant difference (LSD) post hoc test; n = 15 (A–E) or n = 8 (F and G). Error bars indicate the SE. See also Figure S2 and Table S1.

increases in dry weight in comparison to other lines used in this study. In particular, at the middle and base of trees, the dry weight of *PtPP2::PttCLE41-PttANT::PttPXY* stem segments were on average more than twice the weight of the control plants.

In order to ensure that the differences observed were reproducible, we clonally propagated material from six independent *PtPP2::PttCLE41-PttANT::PttPXY* lines. We monitored the growth of these plants weekly, starting shortly after transfer to soil. The diameter of several clones was significantly bigger

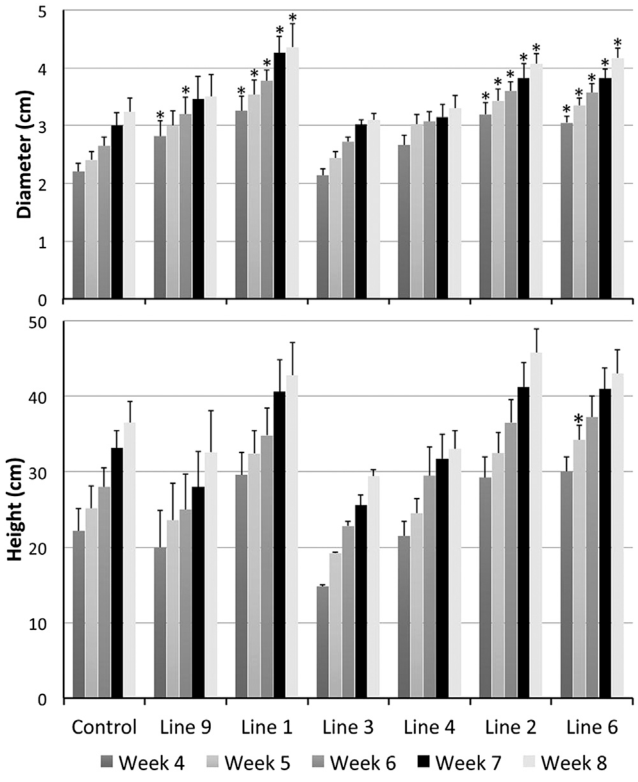

Figure 4. Growth of Clonally Propagated Plants Derived from Independent Transformants of *PtPP2::PttCLE41-PttANT::PttPXY*

Diameter (top) and height (bottom) of plants were measured at weekly intervals starting 4 weeks after transfer from tissue culture to soil. Asterisk indicates a p value of less than 0.05 compared to the controls. All p values were calculated with an ANOVA and a LSD post hoc test; n = 6 for the control; n = 5 for *PtPP2::PttCLE41-PttANT::PttPXY* lines 1, 3, and 9; n = 4 for lines 2 and 4. Error bars indicate the SE. See also Figure S4.

than wild-type at all stages monitored (Figure 4). There was also variation between clones such that plants from line 2 were both significantly taller and exhibited a significantly larger diameter than plants from line 3 at all five time points examined (Figure 4).

Conclusions

Trees represent a huge natural resource used for the production of paper, fuel, and materials and are an increasingly important carbon sink [15] that can help to ameliorate anthropogenic increases in atmospheric CO_2. Recently, trees have also been the focus of intense interest as a renewable source of plant biomass that may be converted into bioethanol [16] and other chemicals for the rapidly expanding field of industrial biotechnology [17]. The majority of biomass in trees is derived from radial growth that is characterized by growth rings in the wood. The size of each growth ring is intimately linked to the environmental conditions during the growing season that year.

Our data suggest that the PXY-CLE pathway functions in trees to regulate secondary growth and is likely to be central to the way in which trees evolved secondary growth. Together, the analysis demonstrates that by engineering the PXY-CLE pathway, we were able to dramatically increase secondary growth in plants shortly after they were first rooted (Figures 3 and S2C), the earliest point they could be analyzed, and the increase in xylem was maintained in plants grown for up to a year (Figures 4 and S2D). These results indicate that this pattern of growth is likely to continue during the lifetime of the tree, thereby providing a means of dramatically increasing tree productivity that would help to meet the increasing demand for renewable resources.

While tree productivity may benefit from anthropogenic increases in atmospheric CO2, climate models and recent changes in weather pattern strongly suggest that we are entering a period in which large parts of the globe experience more frequent exposure to extreme and changeable weather [18] that is likely to have detrimental effects on growth. It will be important to establish whether manipulating PXY-CLE signaling will enable us to override the environmental cues that normally regulate plant growth and thus enable us to generate trees that are able to maintain high productivity even when exposed to more extreme environmental conditions.

ACCESSION NUMBERS

The GenBank accession numbers for PttPXY and PttCLE41 reported in this paper are KP682331 and KP682332, respectively.

SUPPLEMENTAL INFORMATION

Supplemental Information includes Supplemental Experimental Procedures, four figures, and one table and can be found with this article online at http://dx.doi.org/10.1016/j.cub.2015.02.023.

AUTHOR CONTRIBUTIONS

J.P.E., M.K., and S.R.T. designed the experiments. L.S.M., J.P.E., L.C., and M.K. carried out the experimental work. S.R.T., J.P.E., and M.K. wrote the manuscript.

ACKNOWLEDGMENTS

The authors are grateful to Bjorn Sundberg for providing the T89 clone. We thank Joe Ogas, Patrick Gallois, Thomas Nuhse, Stephen High, and Minsung Kim for critical reading of the manuscript. This work was funded by BBSRC (grant number BB/H019928).

Received: October 6, 2014
Revised: December 16, 2014
Accepted: February 5, 2015
Published: April 9, 2015

REFERENCES

1. Miyashima, S., Sebastian, J., Lee, J.Y., and Helariutta, Y. (2013). Stem cell function during plant vascular development. EMBO J. *32*, 178–193.
2. Ursache, R., Nieminen, K., and Helariutta, Y. (2013). Genetic and hormonal regulation of cambial development. Physiol. Plant. *147*, 36–45.
3. Etchells, J.P., and Turner, S.R. (2010). The PXY-CLE41 receptor ligand pair defines a multifunctional pathway that controls the rate and orientation of vascular cell division. Development *137*, 767–774.
4. Fisher, K., and Turner, S. (2007). PXY, a receptor-like kinase essential for maintaining polarity during plant vascular-tissue development. Curr. Biol. *17*, 1061–1066.
5. Hirakawa, Y., Shinohara, H., Kondo, Y., Inoue, A., Nakanomyo, I., Ogawa, M., Sawa, S., Ohashi-Ito, K., Matsubayashi, Y., and Fukuda, H. (2008). Non-cell-autonomous control of vascular stem cell fate by a CLE peptide/receptor system. Proc. Natl. Acad. Sci. USA *105*, 15208–15213.

6. Kondo, T., Sawa, S., Kinoshita, A., Mizuno, S., Kakimoto, T., Fukuda, H., and Sakagami, Y. (2006). A plant peptide encoded by CLV3 identified by in situ MALDI-TOF MS analysis. Science *313*, 845–848.

7. Etchells, J.P., Provost, C.M., Mishra, L., and Turner, S.R. (2013). *WOX4* and *WOX14* act downstream of the PXY receptor kinase to regulate plant vascular proliferation independently of any role in vascular organisation. Development *140*, 2224–2234.

8. Hirakawa, Y., Kondo, Y., and Fukuda, H. (2010). TDIF peptide signaling regulates vascular stem cell proliferation via the WOX4 homeobox gene in Arabidopsis. Plant Cell *22*, 2618–2629.

9. Kondo, Y., Ito, T., Nakagami, H., Hirakawa, Y., Saito, M., Tamaki, T., Shirasu, K., and Fukuda, H. (2014). Plant GSK3 proteins regulate xylem cell differentiation downstream of TDIF-TDR signalling. Nat. Commun. *5*, 3504.

10. Whitford, R., Fernandez, A., De Groodt, R., Ortega, E., and Hilson, P. (2008). Plant CLE peptides from two distinct functional classes synergistically induce division of vascular cells. Proc. Natl. Acad. Sci. USA *105*, 18625–18630.

11. Nilsson, O., Little, C.H.A., Sandberg, G., and Olsson, O. (1996). Expression of two heterologous promoters, Agrobacterium rhizogenes rolC and cauliflower mosaic virus 35S, in the stem of transgenic hybrid aspen plants during the annual cycle of growth and dormancy. Plant Mol. Biol. *31*, 887–895.

12. Schrader, J., Nilsson, J., Mellerowicz, E., Berglund, A., Nilsson, P., Hertzberg, M., and Sandberg, G. (2004). A high-resolution transcript profile across the wood-forming meristem of poplar identifies potential regulators of cambial stem cell identity. Plant Cell *16*, 2278–2292.

13. Tuskan, G.A., Difazio, S., Jansson, S., Bohlmann, J., Grigoriev, I., Hellsten, U., Putnam, N., Ralph, S., Rombauts, S., Salamov, A., et al. (2006). The genome of black cottonwood, Populus trichocarpa (Torr. & Gray). Science *313*, 1596–1604.

14. Carpenter, A.E., Jones, T.R., Lamprecht, M.R., Clarke, C., Kang, I.H., Friman, O., Guertin, D.A., Chang, J.H., Lindquist, R.A., Moffat, J., et al. (2006). CellProfiler: image analysis software for identifying and quantifying cell phenotypes. Genome Biol. *7*, R100.

15. Stephenson, N.L., Das, A.J., Condit, R., Russo, S.E., Baker, P.J., Beckman, N.G., Coomes, D.A., Lines, E.R., Morris, W.K., Rüger, N., et al. (2014). Rate of tree carbon accumulation increases continuously with tree size. Nature *507*, 90–93.

16. Somerville, C. (2006). The billion-ton biofuels vision. Science *312*, 1277.

17. Raunikar, R., Buongiorno, J., Turner, J.A., and Zhu, S. (2010). Global outlook for wood and forests with the bioenergy demand implied by scenarios of the Intergovernmental Panel on Climate Change. For. Policy Econ. *12*, 48–56.

18. Palmer, T. (2014). Atmospheric science. Record-breaking winters and global climate change. Science *344*, 803–804.

Current Biology

Supplemental Information

Wood Formation in Trees Is Increased by Manipulating PXY-Regulated Cell Division

J. Peter Etchells, Laxmi S. Mishra, Manoj Kumar, Liam Campbell, and Simon R. Turner

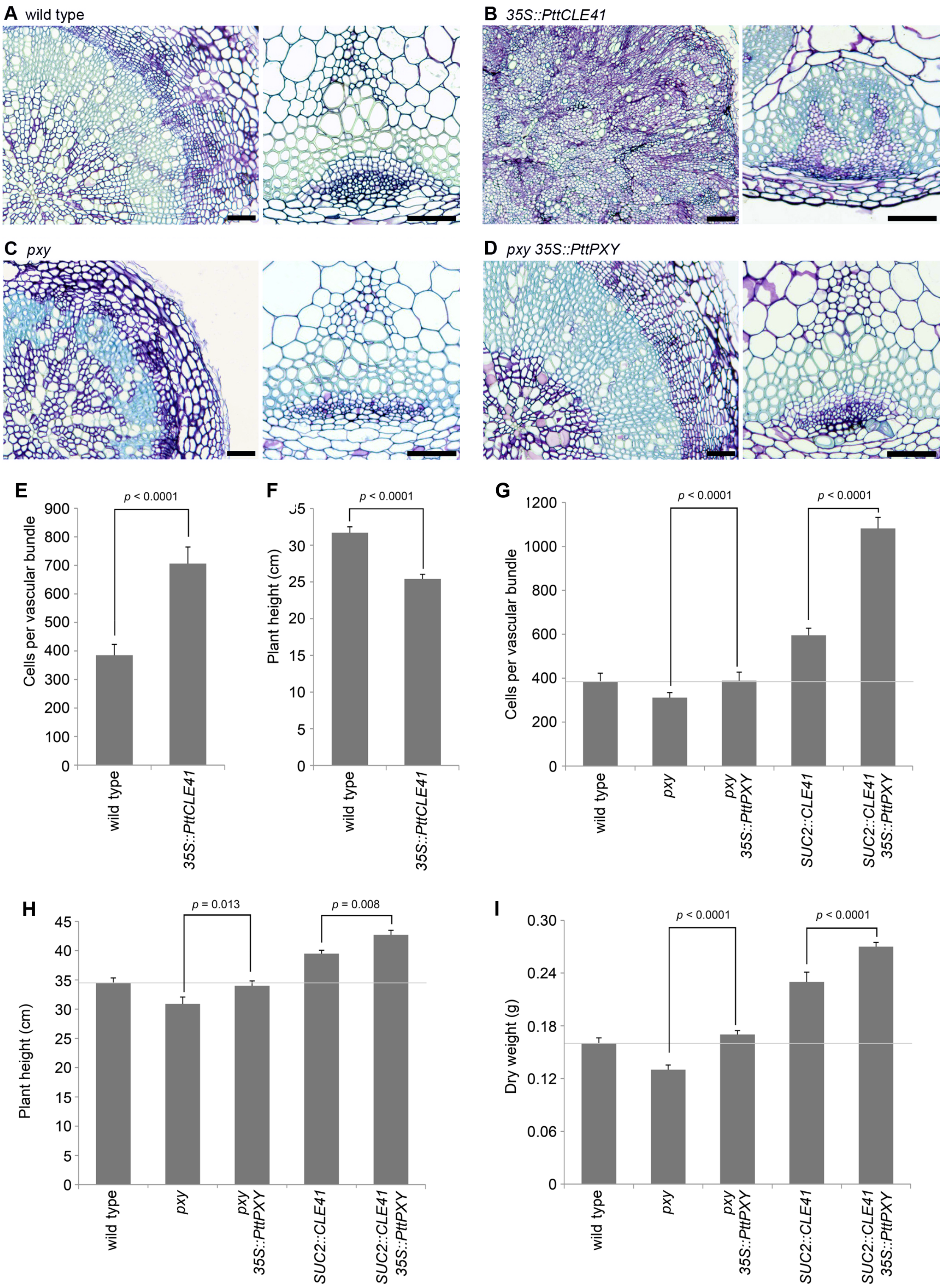

Figure S1: Poplar *CLE41* and *PXY* genes are functional in *Arabidopsis*.
(**A-D**) Sections from *Arabidopsis* hypocotyls (LHS) and inflorescence stem vascular bundles (RHS) from typical wild type (**A**), *35S::PttCLE41* (**B**), *pxy* mutant (**C**), and a *pxy* mutant complemented with *35S::PttPXY* (**D**). **(E-I)** Growth characteristics of *Arabidopsis* lines overexpressing poplar *PXY* genes. Number of cells per vascular bundle (**E**) and plant height (**F**) of *Arabidopsis 35S::PttCLE41* lines compared to wild type counterparts. Number of cells per vascular bundle (**G**), plant height (**H**) and dry weight (**I**) of *Arabidopsis pxy*, *pxy 35S::PttPXY*, *SUC2::AtCLE41*, *SUC2::AtCLE41-35S::PttPXY* lines compared to wild type counterparts. P values were calculated with an ANOVA and LSD post-hoc test with N = 10 (E,F) or 40 (G,H,I). Scales bars indicate 50 μM (A-D). Error bars indicate the standard error. Related for Figure 1.

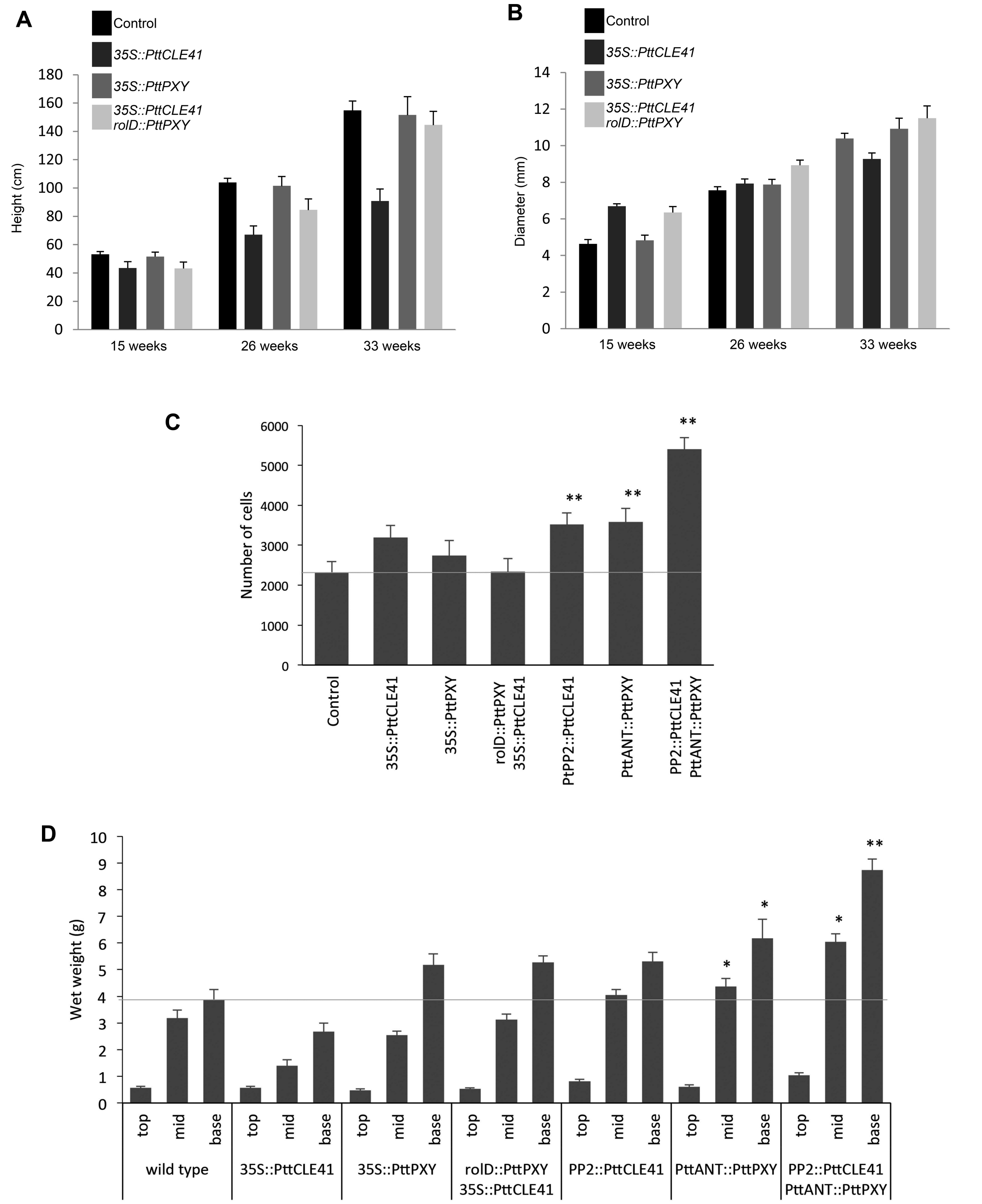

Figure S2: Growth characteristics of aspen lines overexpressing *PttCLE41/PttPXY*. Height (**A**) and diameter (**B**) measurements from hybrid aspen grown in soil. Trees rooted in April, were measured at 15 weeks (July), 26 weeks (August) and 33 weeks (October). (**C**) Graph showing number of vascular cells in control and *35S::PttCLE41*, *35S::PttPXY*, 35S::*PttCLE41 rolD::PttPXY*, *PtPP2::PttCLE41*, *PttANT::PttPXY* and *PtPP2::PttCLE41 PttANT::PttPXY* hybrid aspen lines in tissue culture 3 weeks post-rooting. (**D**) Wet weight of 10 cm pieces of sapling stem taken from the base, middle (50th internode) and top, except for *35S::PttCLE41* that had less than 50 internodes and sections were taken midway between the top and bottom instead. Graph shows the wet weight of stem pieces in Fig. 4G. *Significantly larger than wild type $p < 0.05$; ** Significantly larger than all other lines $p < 0.001$; values were calculated with an ANOVA and LSD post-hoc test, A-B, n = 15; C-D, n=7. Error bars indicate the standard error. Related to Figure 3.

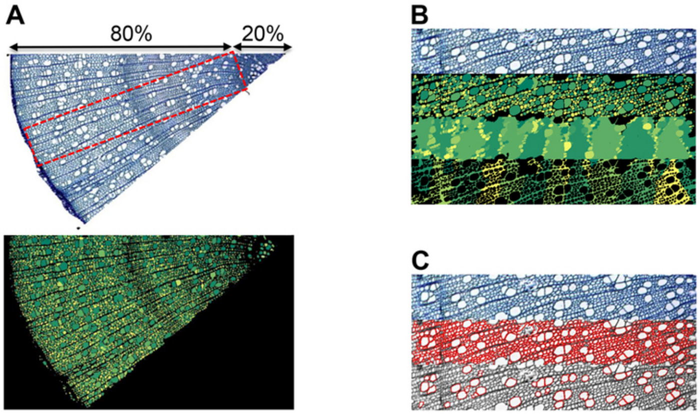

Figure S3: Phenotypic characterisation using Cellprofiler.
(**A**) Transverse stem section from the 50th internode showing xylem in a sector with a central angle of 40° (top) and recognition of cell lumens by Cellprofiler (below). Cells were identified with greater than 95% accuracy, but cells with no clear lumen, such as ray cells or very small fiber cells, were not recognized. (**B**) Measurement of cell size and cell wall area is based upon a rectangle (top) outlined in (a). Primary objects (cell lumens) were identified (upper middle) and propagated outwards to identify the secondary objects (lower middle). The tertiary objects (cell walls) were obtained by subtracting the primary objects from the secondary objects (bottom). (**C**) Identification of vessels based upon identifying primary objects (middle) and then filtering by size and shape (bottom). Relates to the supplementary experimental procedures.

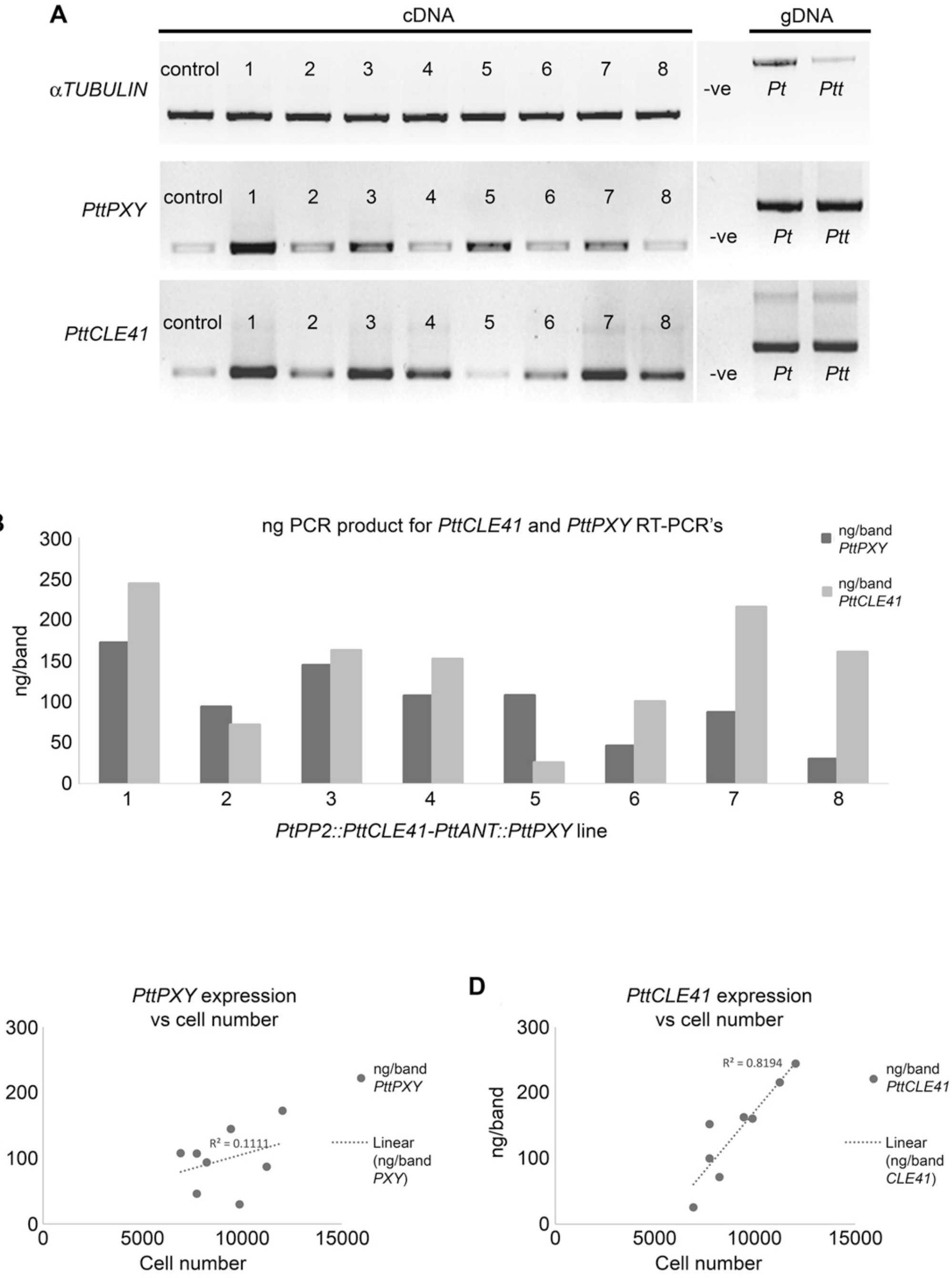

Figure S4: *PttCLE41* and *PttPXY* expression analysis in *PtPP2::PttCLE41-PttANT::PttPXY* lines.

(**A**) RT-PCR showing expression in 8 independent transgenic lines. Stem material was taken adjacent to the 50th internode. (**B**) Relative intensity of PCR product in (A), was determined using Image Lab 5.1 software (Bio-rad). (**C**) Relationship between cell number and *PttPXY* expression. (**D**) Relationship between cell number and *PttCLE41* expression. Related to Figure 4.

Table S1: Analysis of transverse section of xylem from the 50th internode of control and *PtPP2::CLE41-PttANT::PXY* plants.

	Control	*PtPP2::CLE41-PttANT::PXY*
Average cell size	607 ± 13	577 ± 19
Average lumen size	312 ± 13	346 ± 9
Average cell wall area	265 ± 24	260 ± 16
Vessels per 1000 cells	50 ± 2	49 ± 6

Mean of 5 independent lines are shown with the standard error. Area measurements are in arbitrary units. Statistical analysis was carried out using a T-test, no significant differences were found. Related to Figure 4.

Supplementary Experimental Procedures

Binary vectors for plant transformation

For poplar 35S overexpression vectors, primers were designed against *Populus trichocarpa* [S1] *CLE41* (Potri.012G019400) (PttCLE41-F: CACCTAGCTAGCCTTGGTGCTGGT, PttCLE41-R ACCCCTTAATTCCCCCATTA) and *PXY* (Potri.003G107600) (PttPXY-F:CACCATGAAACTCCCTTTTCTTTT, PttPXY-R ACATTCGACTGCAGGCTTTT) and used to amplify sequences from DNA extracted from hybrid aspen (*Populus tremula x tremuloides* clone T89). These were subsequently cloned into pK2GW7 [S2] via pENTR-D-TOPO. For the *rolD::PttPXY 35S::CLE41* construct, *PttCLE41* was subcloned into pDONRP4-P3 which was combined with *pENTR-D-TOPO-PttPXY* and *pK7m34GW2-8m21GW3* [S3] using an LR clonase reaction. Cloned *PttPXY* and *PttCLE41* were sequenced either in entry clones or expression clones. Sequences were annotated by aligning with *P. trichocarpa* sequences. Annotated sequences for *PttPXY* (accession number, KP682331) and *PttCLE41* (accession number, KP682332) were submitted to NCBI. During the cloning a mutation was accidently introduced that had removed the stop codon at the end of the *PttPXY* gene and resulted in a 44 amino acid extension encoded by the vector being added to the C terminus.

For tissue specific expression, *PttPXY* and *PttCLE41* pENTR-D/TOPO entry clones were used in an LR clonase reaction in combination with custom Gateway destination vectors, pVX31 (*Apa*I-p*PttANT1*-*Spe*I-R1R2 Gateway Cassette-t35S-*Sbf*I) and pVX33 (*Sbf*I-p*PtPP2*-*Spe*I-R1R2 Gateway Cassette- t35S-*Sbf*I), which were constructed in a pCambia2300 backbone using restriction based cloning. The promoter sequences were chosen on the basis of poplar expression data. *PttANT* (Potri.002G114800) regulatory sequences were used for cambium specific expression and a *PtPP2* (Potri.015G120200) promoter was used for phloem specific expression. For the *PtPP2* promoter from *Populus trichocarpa* primers (pPtPP2-F atccctaggcctgcaggTAAGCTATGTACGTTTTGG, pPttANT-R atcactagtGACAAGCTGAGAGACTG) were used to amplify a fragment of 1999 bp upstream of the start codon. For the *PttANT1* promoter, primers (pPttANT-F atcgggcccCCGAAGTTGCTCACTTC, pPttANT-R atcactagtGACAAGCTGAGAGACTG) were used to amplify a sequence 1156 bp upstream of start codon to 904 bp downstream of the start codon that drove expression in vascular tissue. To create the double tissue specific expression construct, *Sbf*I-*pPtPP2-PttCLE41-* t35S-*Sbf*I cassette was excised and cloned into *Sbf*I site of *Apa*I-*pPttANT1-PttPXY*-t35S-*Sbf*I.

Transcriptional reporter lines for p*PttANT* and p*PtPP2* were generated by cloning a fragment encoding eGFP-GUS in in pVX31 and pVX33 using an LR clonase reaction resulting in p*PttANT::eGFP-GUS* and p*PtPP2::eGFP-GUS* constructs. Hand sections were stained using a variation on the method described in Rodrigues- Pousada [S4] and were viewed following clearing by overnight incubation at 4°C in chloral hydrate solution [S5].

Plant transformation

Arabidopsis transformation was carried out using the method of Clough and Bent [S6]. For transformation of hybrid aspen (clone T89), a method based on that of Nilsson et al [S7] was used. Briefly, *Agrobacterium* strain GV3101 harbouring a binary vector was grown to an OD_{600} of 0.6, collected by centrifugation in a 50 ml tube and resuspended in MS medium, pH5.8 supplemented with acetosyringone to a final concentration of 25 μM at room temperature. Leaf and petiole sections were cut

from hybrid aspen grown under sterile conditions and incubated in the resuspended *Agrobacterium* for 1 hour and placed on MS agar supplemented with 2% sucrose, BAP (0.2 mg/L), IBA 0.1 mg/L and TDZ (0.1 mg/L) prior to incubation in dark for 48 hours. Subsequently, plant pieces were rinsed in MS and placed in the light on MS agar supplemented with 250 µg/ml cefatoxime and 100 µg/ml kanamycin. Following shoot initiation calli were transferred to woody plant medium (WPM) supplemented with sucrose (2%), BAP (0.2 mg/L), IBA (0.1 mg/L), kanamycin (100 µg/ml) for shoot elongation and subsequently to WPM for rooting.
In order to synchronise plant growth for subsequent analysis the top 2 cm of each plantlet to be used was removed and re-rooted on the same day. All plants used for growth analysis were grown side by side in the same incubator and transferred to soil on the same day once roots were established. For long term growth, plants were transferred to a greenhouse in April and maintained for up to 12 months.

Determination of plant growth characteristics
Vascular organization was determined using plant material fixed in FAA, dehydrated through an ethanol series before infiltration and embedding with JB4 embedding media. 5 µM sections were stained with 0.05% aqueous toluidine blue, mounted in Cytoseal and visualised on a Leica 5500 microscope. Vascular tissue was considered to be ordered if xylem could be incorporated in an elliptical shape that excluded the phloem. Xylem cell counts and determination of cell wall area was performed on cross sections from the entire cross section of tissue culture plants 3 weeks post rooting or from the 50th internode of greenhouse grown plants in which case only a 40° segment of the stem was used (Figure S3A). Cell counting was carried out using Cellprofiler [S8] as outlined in Figure S3. For greenhouse grown plants, 10cm segments were sampled from base of the plant, from 50th internode and from the top of the plants, 12 months following transfer to soil. Material was dried at 50°C for 4 weeks before weighing.

Supplementary references

S1. Tuskan, G.A., DiFazio, S., Jansson, S., Bohlmann, J., Grigoriev, I., Hellsten, U., Putnam, N., Ralph, S., Rombauts, S., Salamov, A., et al. (2006). The Genome of Black Cottonwood, Populus trichocarpa (Torr. & Gray). Science *313*, 1596-1604.
S2. Karimi, M., Inze, D., and Depicker, A. (2002). GATEWAY(TM) vectors for Agrobacterium-mediated plant transformation. Trends in Plant Science *7*, 193-195.
S3. Karimi, M., Bleys, A., Vanderhaeghen, R., and Hilson, P. (2007). Building Blocks for Plant Gene Assembly. Plant Physiology *145*, 1183-1191.
S4. Rodrigues-Pousada, R.A., De Rycke, R., Dedonder, A., Van Caeneghem, W., Engler, G., Van Montagu, M., and Van Der Straeten, D. (1993). The Arabidopsis 1-Aminocyclopropane-1-Carboxylate Synthase Gene 1 Is Expressed during Early Development. The Plant Cell Online *5*, 897-911.
S5. Berleth, T., and Jurgens, G. (1993). The role of the monopteros gene in organising the basal body region of the Arabidopsis embryo. Development *118*, 575-587.
S6. Clough, S.J., and Bent, A.F. (1998). Floral dip: a simplified method for Agrobacterium-mediated transformation of Arabidopsis thaliana. Plant Journal *16*, 735-743.
S7. Nilsson, O., Aldén, T., Sitbon, F., Anthony Little, C.H., Chalupa, V., Sandberg, G., and Olsson, O. (1992). Spatial pattern of cauliflower mosaic

virus 35S promoter-luciferase expression in transgenic hybrid aspen trees monitored by enzymatic assay and non-destructive imaging. Transgenic Research *1*, 209-220.

S8. Carpenter, A.E., Jones, T.R., Lamprecht, M.R., Clarke, C., Kang, I.H., Friman, O., Guertin, D.A., Chang, J.H., Lindquist, R.A., Moffat, J., et al. (2006). CellProfiler: image analysis software for identifying and quantifying cell phenotypes. Genome Biol *7*, R100.
